# Atypical Parkinsonism: An Uncommon Presentation of Disseminated Coccidioidomycosis

**DOI:** 10.7759/cureus.67676

**Published:** 2024-08-24

**Authors:** Amy H Sim, Naila Kausar, Jessica J Garcia-Chan, Thomas O´neill, Sushma Yerram

**Affiliations:** 1 Neurology, Texas Tech University Health Sciences Center El Paso, El paso, USA; 2 Neurology, Texas Tech University Health Sciences Center El Paso, El Paso, USA; 3 Neuroradiology, Texas Tech University Health Sciences Center El Paso, El paso, USA

**Keywords:** cerebral infarction, vasculitis, gait abnormalities, atypical parkinsonism, coccidioidal meningitis, disseminated coccidioidomycosis

## Abstract

Coccidioidomycosis is endemic in the southwestern United States, Central America, and South America. Coccidioidomycosis has a variety of clinical presentations. Coccoidal meningitis is a feared form of disseminated coccidioidomycosis with high mortality and mobility rates. We reported a case of a 64-year-old man who presented with a three-week history of gait abnormalities and back pain. The patient had atypical parkinsonism, signs of cogwheeling rigidity, a masked face, intention tremor, a shuffling gait, upgazed restriction, and long track signs of left Babinski. MRI of the brain and cervical spine demonstrated scattered foci of abnormal parenchymal and leptomeningeal enhancement. The patient later developed acute cerebral infarction before a definite diagnosis of disseminated coccidioidomycosis, which was made when the result was that serum and cerebrospinal fluid coccidioidomycosis antibodies were high. The patient started lifelong antifungal treatment. We provide a natural disease process from atypical parkinsonism to cerebral infarction to hydrocephalus to enhance awareness of the myriad clinical presentations, emphasize the importance of endemic mycoses awareness, and also put forward a question of what can be done to detect coccidioidomycosis early.

## Introduction

Coccidioidomycosis is a dimorphic fungal infection transmitted by inhalation of airborne spores of soil-dwelling *Coccidioides spp.* organisms, such as *C. immitis* or *C. posadasii*. It is mainly infected in the summer or late fall. It is endemic in the southwestern United States and Central and South American regions, with Arizona and California being hyperendemic states [1]. There were 17,612 cases of coccidioidomycosis in the US reported to the CDC and 7459 new cases in California in 2022. There were three thousand eighty-nine coccidioidomycosis-associated deaths in the United States during 1990-2008 [2]. The number is underestimated, and many patients are not tested for coccidioidomycosis. 54% of patients with coccidioidomycosis visited healthcare providers more than three times before being tested for coccidioidomycosis, 70% were diagnosed with another disease, and 83% were prescribed antibacterial medication [3].

Coccidioidomycosis has myriad clinical presentations, from asymptomatic infection to mild, self-limited flu-like symptoms, severe pulmonary manifestations, meningitis, and fatal outcomes. Approximately 60% of infected individuals remain asymptomatic, while 1% develop disseminated disease involving the skin, joints, bones, central nervous system, and other organs. Filipino or African ethnicity, the elderly, pregnant women, the immunocompromised population (HIV/AIDS, immunosuppressive medications, etc.), diabetes, transplant recipients, and prisoners are at high risk for severe or disseminated coccidioidomycosis [4]. We are presenting a case of dissemination coccidioidomycosis with coccidioidomycosis meningitis with an initial atypical presentation and complication with cerebral infarction and hydrocephalus, subsequently to improve awareness of coccidioidomycosis meningitis and prompt early diagnosis and treatment of coccidioidomycosis.

## Case presentation

A 64-year-old male with a past medical history of diabetes and hypertension presented with back pain and gait imbalance for three weeks. Two days before the gait issue, the patient had intermittent occipital shooting pain in the head, which subsided three hours after he took Tylenol. After getting off the flight from the US to South Korea for his vacation, he felt back pain, and he had to drag his legs to ambulate. Due to a worsening gait problem, the patient fell on the left side, so the daughter took the patient to seek health care. The patient did an MRI of the lumbar spine, which revealed some disc bulging but did not mention any spinal cord compression. The details of the clinical course are unknown. Also, the patient noted a decreased pitch of his voice, slower speech, and bilateral upper extremity intention tremor. He denied any preceding upper respiratory infection or gastrointestinal tract illness. A physical exam on admission showed that the patient was intact in mental status, language, speech, and cranial nerves except for hypophonia and a masked face. The patient had left upper extremity rigidity, intention tremors, and normal strength. Light touch, proprioception, pinprick, and temperature sensation tests were normal. Deep tendon reflexes were normal. There was a Babinski sign on the left side, a positive jaw jerk. The rapid alternating movement was slower and denser on the left than on the right, with decreased amplitude on finger tapping. There was a shuffling gait with decreased arm swings and a slow turn. Romberg was positive.

**Figure 1:**
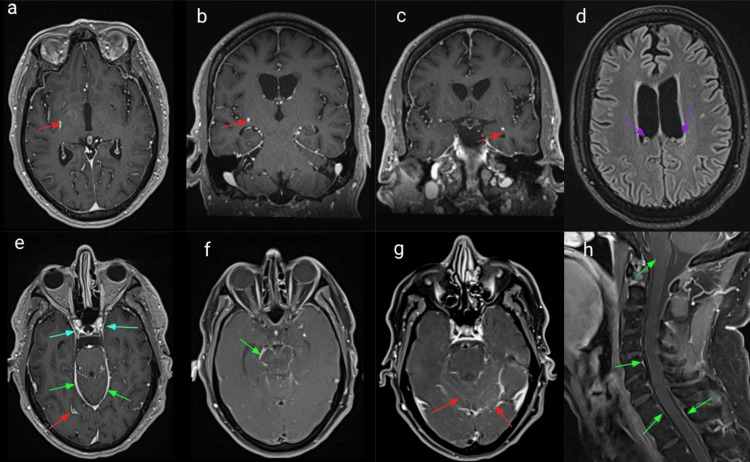
Magnetic resonance imaging (MRI) of the of the brain and cervical spine with and without contrast T1-weighted images in the axial (a, e, f, g), coronal (b, c), and sagittal (h) planes demonstrate scattered foci of abnormal parenchymal enhancement in the right globus pallidus (a: red arrow) and posterolateral putamen (b: red arrow), lateral aspect left uncus/temporal stem (c: red arrow), right occipital cortex (e: red arrow), as well as diffuse leptomeningeal enhancement along the basilar region (e: green arrow), the midbrain (f: green arrow), the cerebellar folia (g: green arrow) and several bilateral cranial nerves (e: blue arrow) and spinal cord (h: green arrow). Multiple subependymal nodules along the lateral ventricles with an increased T2 fluid-attenuated inversion recovery (FLAIR) signal (d: purple arrow).

Given the rapid development of parkinsonism symptoms with the concomitant long tact sign of left-side Babinski, an extensive workup was performed. Initial serological lab work is unremarkable except for the hyponatremia (Table 1). A lumbar puncture was performed, and cerebrospinal fluid (CSF) analysis showed a total nucleated cell of 290 cells/ml, elevated segmented neutrophils percentage (5%), protein level (300 mg/dL), and hypoglycorrhachia (34 mg/dL), but red blood cells, lymphocyte percentage in CSF (Lymph CSF %), macrophage percentage, Venereal Disease Research Laboratory (VDRL), meningitis panel, encephalitis antibodies (Abs), West Nile antibody immunoglobulin G (Ab IgG), and immunoglobulin M (IgM) are unremarkable (Table 2). Magnetic resonance imaging (MRI) brain and cervical spine with and without contrast (Figure 1) showed that T1-weighted images in axial (a, e, f, g) and coronal (b, c) and sagittal (h) planes demonstrate scattered foci of abnormal parenchymal enhancement in the right globus pallidus (a: red arrow) and posterolateral putamen (b: red arrow), lateral aspect left uncus/temporal stem (c: red arrow), right occipital cortex (e: red arrow), as well as diffuse leptomeningeal enhancement along basilar region (e: green arrow), the midbrain (f: green arrow), the cerebellar folia (g: green arrow) and several bilateral cranial nerves (e: blue arrow) and spinal cord (h: green arrow). Multiple subependymal nodules along the lateral ventricles with an increased T2 fluid-attenuated inversion recovery (FLAIR) signal (d: purple arrow). CT thorax with contrast showed a 5.4 mm nodule in the right upper lobe, which was not specific and most likely benign on imaging. Also, the patient did not have any lung manifestations.

**Table 1:** Serological lab work The patient has hyponatremia with a sodium (Na) level of 128 mmol/L, and the *Coccidioides* antibody titer level is high at 1: 32; otherwise, the erythrocyte sedimentation rate (ESR), C-reactive protein (CRP), rapid plasma reagin (RPR), methylmalonic acid, human immunodeficiency virus (HIV)1/2, paraneoplastic profile, brucella immunoglobulin M (IgM), brucella immunoglobulin G (IgG), encephalitis antibodies (Abs), West Nile antibody immunoglobulin G (Ab IgG), and immunoglobulin M (IgM) are unremarkable.

Serum	Patient Value	Reference range
Na	128	<135-145mmol/L
ESR	2	0-19mm/hr
CRP	0.03	0-1mg/dL
RPR	Non-reactive	N/A
Methylmalonic acid	102	87-318nmol/L
HIV 1/2	Negative	N/A
Paraneoplastic profile	Negative	N/A
Brucella IgM	0.2	< 0.8 not detected
Brucella IgG	<0.13	< 0.13 not detected
Encephalitis Abs	Negative	N/A
West Nile Ab IgG	<1.3	<1.3 not detected
West Nile Ab IgM	<0.9	<0.9 not detected
Coccidioides Ab	1:32High	<1:2

**Table 2:** Cerebrospinal fluid analysis Total nucleated cells, protein, and segmented neutrophils percentage in the cerebrospinal fluid (SEG CSF%) are elevated, 290/uL, more than 300mg/dL, and 5%, respectively. The glucose level is slightly low at 34 mg/dL, and the and the *Coccidioides* antibody (*Coccidioides* Ab) titer level is high at 1: 32; otherwise, red blood cells, lymphocyte percentage in CSF (Lymph CSF %), macrophages, Venereal disease research laboratory (VDRL), meningitis panel, encephalitis antibodies (Abs), West Nile antibody immunoglobulin G (Ab IgG), and immunoglobulin M (IgM) are unremarkable.

CSF	Patient Value	Reference range
Total nucleated cells	290	0-5/uL
RBC	3	0-5/uL
Glucose	34	40-70mg/dL
Protein	>300	12-60mg/dL
SEG CSF%	5%	0-2%
Lymph CSF%	85%	63-99%
Macrophage CSF %	10%	3-37%
VDRL	Non-reactive	N/A
Meningitis panel	Negative	N/A
Encephalitis Abs	Negative	N/A
West Nile Ab IgG	<1.3	<1.3 not detected
West Nile Ab IgM	<0.9	<0.9 not detected
Coccidioides Ab	1:32High	<1:1

However, the patient’s Parkinsonism symptoms subsided, and gait abnormality improved during the hospitalization while still waiting for more workup results. *Coccidioides* antibody titers later proved high both in serum and CSF. The patient did not have other signs of infection to prompt the start of antibiotics. The patient was discharged and instructed to follow up at the clinic.

**Figure 2:**
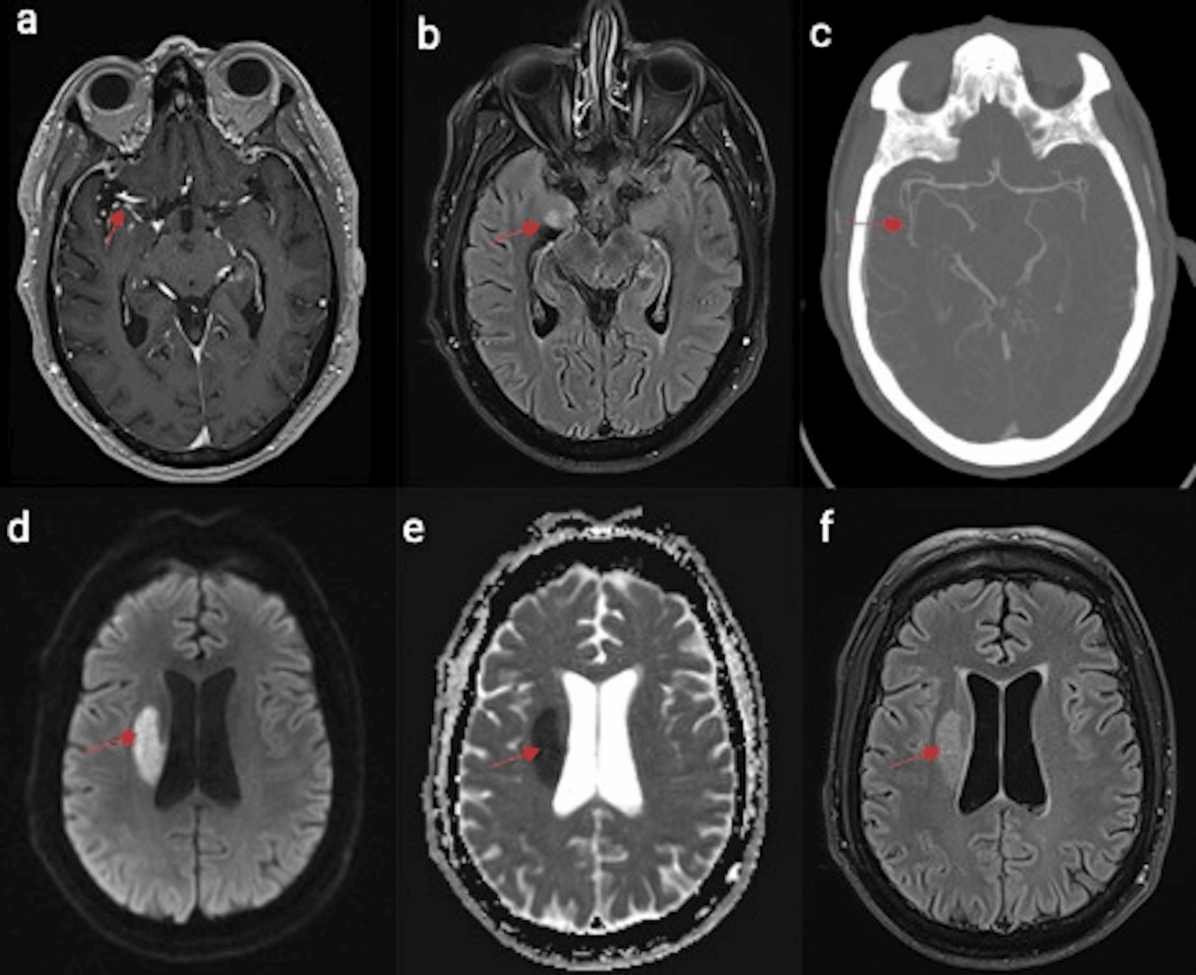
Magnetic resonance imaging (MRI) of the brain and computed tomography angiography (CTA) of the brain Diffusion-weighted imaging (DWI) hyperintensity (d: red arrow)/decreased apparent diffusion coefficient (ADC) (e: red arrow) and increased fluid-attenuated inversion recovery (FLAIR) signal (f: red arrow) in the right corona radiata, putamen, and amygdala (d, e, f, b) correspond to likely a secondary perforating infarct with foci of adjacent leptomeningeal enhancement along the lenticulostriate perforators (a: red arrow). CTA demonstrates right proximal M2 narrowing (c: red arrow).

Unfortunately, the patient came back in three days with slurred speech, left facial weakness, and left-side weakness. On exam, the patient was dysarthric and had mild left facial weakness, minimal drift in the left side with arm rolling favoring the right arm, increased tone in all four extremities, and an ataxic gait. The NIH stroke scale was 4. A CT scan shows a subacute infarct in the right insular cortex and lateral aspect of the putamen on the right side. Magnetic resonance imaging (MRI) brain demonstrated diffusion-weighted imaging (DWI) hyperintensity (d: red arrow)/decreased apparent diffusion coefficient (ADC) (e: red arrow) and increased fluid-attenuated inversion recovery (FLAIR) signal (f: red arrow) in the right corona radiata, putamen, and amygdala (d, e, f, b), corresponding to likely a secondary perforating infarct with foci of adjacent leptomeningeal enhancement along the lenticulostriate perforators (a: red arrow). Computed tomography angiography (CTA) of the brain demonstrates right proximal M2 narrowing (c: red arrow) (Figure 2).

After the stroke, the patient lost follow-up due to the patient being admitted to another hospital for endocarditis due to *Enterobacter cloacae*, receiving six weeks of gentamicin and ceftriaxone, and repeated TEE, which was negative for vegetation. Three months later, the patient developed hydrocephalus, and he came to our hospital. Finally, he received antifungal therapy with a high dose of fluconazole.

## Discussion

Coccidioidomycosis mainly occurs in residents of endemic regions, but any travelers to endemic areas can be infected, predominantly agricultural or construction workers in the fall season. In most patients with coccidioidal infection, the primary infection is in the lung, and 0.6% of the infections spread outside of the lungs through either hematogenous or lymphatic spread. Symptoms of primary coccidioidal pneumonia usually occur approximately 7 to 21 days after exposure. Lung lesions are significantly more frequent in the right lung than in the left lung [5]. Patients can have a cough, fatigue, shortness of breath, fever, chest pain, headache, joint pain, and muscle pain. It spreads homogeneously to the meninges and develops coccidioidal meningitis, which is a feared and lethal complication. CM is mainly located in the basilar meninges and can also be located in the lateral cerebral ventricles. 25% of patients with CM have initial respiratory infectious symptoms and systemic symptoms such as fever, anorexia, and generalized weakness [6]. The most common symptoms of CM are persistent headache, photophobia, nausea, vomiting, altered mental status, cognitive decline, or lumbosacral back pain as a result of the lumbar meninges involved. A focal neurology deficit was found at 33%, which may result from vasculitis or a mass lesion. In our case, the patient presented with Parkinsonism because of the involvement of the basal ganglia. Tremulousness and intention tremors, gait abnormalities, and ataxia are sometimes present physically [7]. However, nuchal rigidity and Kernig and Brudzinski signs are absent. Unfortunately, upon presentation with meningitis, there may be few or no symptoms associated with the primary respiratory infection, or even the radiographic study is not apparent; the patient did not have a fever or headache but had back pain reflecting the lumbar meninges, as in this case. The patient did not have a fever or headache but had back pain reflecting the lumbar meninges. The patient lived in El Paso, which was also an endemic region, so coccidioidal meningitis was suspected.

Complications of coccoidal meningitis include hydrocephalus, cerebral infarction, spinal arachnoiditis, cerebral abscess, vertebral artery aneurysm, multiple intracranial aneurysms (up to 10 aneurysms) [8], vasospasm, adhesive arachnoiditis, and a spinal cord syrinx. Post-infection has inflammatory changes of small and middle-sized cerebral arteries and veins, resulting in vascular insufficiency and focal neurologic deficit mimicking stroke, and the transmural inflammatory process to the vessel lumen results in thrombosis [9]. Vasculitis with encephalitis and coccidioidal meningitis are reported [10]. 

Nonspecific laboratory abnormalities of coccidological meningitis may include hyponatremia associated with the syndrome of inappropriate antidiuretic hormone. The diagnosis criteria require lumbar cerebrospinal fluid analysis demonstrating lymphocytic pleocytosis, hypoglycorrhachia, and elevation of protein up to 250 mg/dL with the established anti-coccidioidal antibodies in the CFS. To diagnose an early coccidioidal infection, enzyme immunoassays (EIAs) for anticoccidial immunoglobulin M (IgM) and immunoglobulin G (IgG) are commercially available, and they are twice as sensitive as standard immunodiffusion-based tests for traditional tube precipitin (IDTP) or complement-fixing (IDCF) anticoccidial antibodies [11].

Amphotericin B and oral triazoles are the main antifungal therapies for coccidioidomycosis. Intravenous amphotericin B was the first effective therapy used in 1957. Since the 1980s, various oral antifungal agents have emerged and taken over the leading role in coccidioidomycosis management. The two-year mortality rate of CM was 100% if untreated [5]. For patients with newly diagnosed CM, according to the 2016 Infectious Diseases Society of America Clinical Practice Guidelines, the first line of primary treatment is fluconazole 400-1200 mg orally daily for patients with normal renal function or itraconazole 200 mg every 12 hours with fatty food and an acidic beverage to increase absorption. Amphotericin B is reserved for refractory cases as rescue therapy in azole failure [12]. The duration of treatment for the infection is often prolonged and may last several months to years, or even lifelong, to prevent relapse. Coccoidal meningitis requires lifelong treatment. A retrospective cohort study of adjunctive corticosteroid therapy in coccidioidal meningitis significantly reduced secondary cerebrovascular events. However, corticosteroid therapy is still controversial.

## Conclusions

Coccidioidomycosis has protean manifestations, and the mortality and morbidity rates are high. The prognosis depends on the early recognition and treatment of the disease. Recognizing the varied clinical manifestations, endemic regions, risk factors, diagnostic challenges, and therapeutic modalities is crucial. We provide a natural disease process, from gait abnormalities to cerebral infarction, endocarditis, and hydrocephalus, to enhance awareness of the myriad clinical presentations, emphasize the importance of endemic mycoses awareness, and also put forward a question of what can be done to detect the infection, especially in the underserved region, early. Overall, our educational case contributes to the existing literature on disseminated coccidioidomycosis.
